# Microwave-Assisted Synthesis of Pt-Modified NaNbO_3_ Nanowires for Enhanced Photocatalytic Hydrogen Production

**DOI:** 10.1021/acsomega.5c08475

**Published:** 2025-12-05

**Authors:** Marcos R. S. Vicente, Priscila H Palharim, Gabriela T. M. Xavier, Wagner A. Carvalho, Hynd Remita, Juliana S. Souza

**Affiliations:** † 74362 Universidade Federal do ABC, Avenida dos Estados, 5001, Santo Andre, São Paulo 09210580, Brazil; ‡ 27048 Universite Paris-Saclay, Gif-sur-Yvette, Île-de-France 91190, France

## Abstract

The urgent need to address global energy demands and mitigate climate change has driven research toward sustainable methods of hydrogen production. Photocatalytic water splitting represents a promising pathway for the generation of green hydrogen, provided that efficient, stable, and sustainably produced photocatalysts are developed. In this work, orthorhombic sodium niobate (NaNbO_3_) nanowires were synthesized via a microwave-assisted hydrothermal route, a green, time-efficient, and energy-saving strategy that yielded high-surface-area structures. Platinum was incorporated as a cocatalyst to enhance charge separation and promote hydrogen evolution reaction (HER) activity. Comprehensive structural, morphological, and spectroscopic analyses revealed that Pt incorporation occurred at the molecular level, potentially altering the crystal growth orientation, increasing the surface area, and altering the band structure. While Pt loading reduced NaNbO_3_ activity toward the oxygen evolution reaction (OER), it significantly boosted HER performance, achieving 1.26 mmol h^–1^ g^–1^ of H_2_, over four times higher than pristine NaNbO_3_, and comparable to the best reported systems, but obtained through a more sustainable synthesis route. These results highlight the combined benefits of microwave-assisted synthesis and Pt integration in the production of efficient photocatalysts for renewable hydrogen generation.

## Graphical abstract

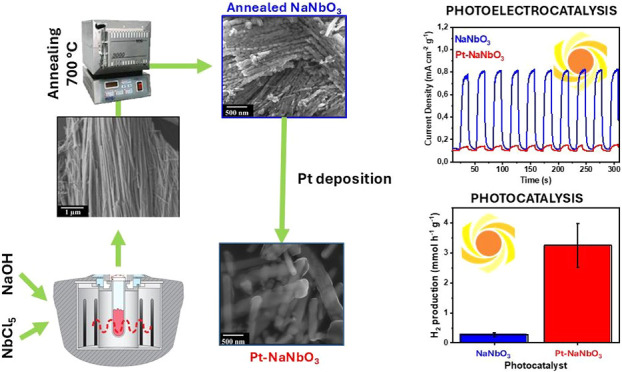

## 1 Introduction

Critical environmental challenges, such as global warming, air pollution, and resource depletion, have necessitated a shift toward sustainable and renewable energy sources.^1,2^ Among the promising alternatives, hydrogen stands out due to its high energy density and zero carbon emissions when used as a fuel. Yet, most of the hydrogen produced today, gray, blue, or brown, relies on nonrenewable processes like natural gas reforming and coal gasification, which emit significant amounts of carbon dioxide. Green hydrogen, produced from renewable sources, offers a cleaner alternative, but its adoption is limited by high energy requirements and costs.^3,4^ A desirable solution is to directly convert solar energy into hydrogen via photocatalysis, which could provide a more sustainable and energy-efficient pathway for green hydrogen production. The success of this approach depends on the development of advanced photocatalysts that can efficiently absorb sunlight and drive the water-splitting reaction.^5,6^

Perovskite semiconductor materials are exciting due to their unique light absorption properties and narrow bandgap, making them well suited for photocatalytic applications, including the degradation of pollutants, CO_2_ reduction, and hydrogen production.^7^ Sodium niobate (NaNbO_3_), a perovskite oxide, is particularly promising because of its high chemical stability, nontoxicity, and potential in photocatalysis. Nonetheless, its photocatalytic efficiency is hindered by rapid recombination of photogenerated charge carriers, which limits its efficiency under visible light.^2,8,9^ To overcome these limitations, strategies such as loading it with noble metals, like platinum (Pt), have been shown to further enhance photocatalytic activity by trapping electrons and improving charge carrier separation.^10–12^ Also, platinum is known to be one of the most effective cocatalysts for the hydrogen evolution reaction.^12,13^

Beyond catalytic performance, the synthesis method should align with sustainable principles. Microwave-assisted synthesis has gained increasing attention for its rapid and uniform heating, significantly shorter reaction times, reduced energy consumption, and enhanced control over the morphology and crystallinity. Microwave energy selectively interacts with the reactive mixture, which consists of precursors and reaction intermediates with varying dielectric constants. These components absorb microwave energy and convert it to heat through molecular rotations caused by the reorientation of dipoles in the electric field. This mechanism facilitates easier bond dissociation and recombination, thereby increasing the overall reaction efficiency.^14,15^ These advantages make microwave synthesis particularly well suited for fabricating nanostructured materials with precise morphological control. Still, its application to NaNbO_3_-based photocatalysts remains limited, as most studies rely on slower, energy-intensive solid-state or hydrothermal methods. Leveraging microwave synthesis could not only improve energy efficiency but also enable uniform Pt deposition on NaNbO_3_ nanowires, two factors critical for enhancing charge separation and catalytic performance.

Although Pt loading has been widely employed to enhance the photocatalytic activity of various semiconductors, its integration with NaNbO_3_ nanowires remains scarcely explored, particularly by using rapid and energy-efficient synthesis routes. Most reported NaNbO_3_-based photocatalysts are prepared via conventional solid-state or hydrothermal methods,^16–18^ which require long reaction times and high energy input, and offer limited control over nanowire morphology and cocatalyst dispersion. Moreover, the influence of Pt modification on the relative efficiencies of the oxygen and hydrogen evolution reactions for NaNbO_3_ has not been fully explored.

In this work, orthorhombic NaNbO_3_ nanowires were synthesized via a microwave-assisted route and subsequently modified with Pt to optimize their photocatalytic properties. The materials were comprehensively characterized and evaluated for both the OER and HER. While Pt incorporation decreased OER activity, it significantly enhanced the HER performance, highlighting the dual catalytic behavior of Pt-modified NaNbO_3_ and underscoring the potential of microwave synthesis for rapid fabrication of high-performance photocatalysts.

## 2 Materials and Methods

### 2.1 Chemicals

All chemicals used were of analytical grade and were used without further purification. Niobium­(V) chloride (NbCl_5_, 99.9%), sodium hydroxide (NaOH, ACS), hexachloroplatinic acid (H_2_PtCl_6_·H_2_O, 99.9%), triethanolamine (TEOA, ≥99%, GC), fluorine-doped tin oxide (FTO) glass (7Ω/sq), and 1-propanol (99.5%) were obtained from Sigma-Aldrich.

### 2.2 Synthesis

#### 2.2.1 NaNbO_3_

The synthesis procedure of NaNbO_3_ was adapted from Fernandes et al.^19^ Briefly, 20 mL of a 10 mol L^–1^ NaOH aqueous solution was mixed with 2.70 g of NbCl_5_ and stirred for 30 min. The mixture was then transferred to a sealed silicon carbide vial with a 30 mL capacity, which supported pressures up to 200 psi. The vial was subjected to a microwave-assisted hydrothermal treatment using a Monowave 400R microwave reactor (Anton-Paar) at 150 °C for 60 min. During irradiation, a constant temperature was kept with a maximum power of 850 W and a heating rate of 4 °C min^–1^. After the reaction, the resulting solid was filtered and washed with Milli-Q water until the supernatant reached a pH of 7.0. The collected solid was dried at 80 °C for 24 h, followed by calcination at 700 °C for 2 h at a heating rate of 10 °C min^–1^.

#### 2.2.2 Incorporation of Pt

NaNbO_3_ was decorated with Pt nanoparticles according to procedures described elsewhere.^20,21^ Briefly, a suspension containing 100 mg of NaNbO_3_ in 20 mL of a 20% (v/v) methanol/water mixture was prepared under magnetic stirring. H_2_PtCl_6_·H_2_O was added at a nominal mass fraction of 0.5% to NaNbO_3_. The mixtures were stirred at 500 rpm and placed 40 cm away from a 500 W mercury lamp (E-40) mounted on a metalized reflector (Taschibra). After 1 h of irradiation, the solid was filtered, washed three times with Milli-Q water, and dried in an oven at 60 °C for 24 h.

### 2.3 Characterization

Powder X-ray diffraction (PXRD) analyses were conducted using a STADI-P diffractometer (Stoe, Darmstadt, Germany) operating at room temperature, 40 mA, 50 kV, and with Cu Kα_1_ (λ = 1.5406 Å) radiation. X-ray diffraction (XRD) profiles were obtained between 2θ values of 10° and 60°. The surface area, pore distribution, and total pore volume were assessed by nitrogen adsorption and desorption isotherms. Approximately 100 mg of the catalysts were degassed for 4 h at 200 °C. A Quantachrome Autosorb −1 MP instrument at −196 °C was used for N_2_ adsorption and desorption measurements. The Brunauer–Emmett–Teller (BET) method was employed to calculate the surface area, and the total pore volume was determined at a relative pressure of *P*/*P*_0_ = 0.98. Thermogravimetric analyses (TGA), a Q500 thermogravimetric analyzer from TA Instruments was used with 15 mg of solid samples, without pretreatment, at a heating rate of 10 °C min^–1^ until reaching 700 °C. Differential scanning calorimetry (DSC) measurements were carried out using a DSC3 differential scanning calorimeter from Mettler Toledo. A T64000 triple Raman spectrometer (Horiba Jobin-Yvon) with excitation at 532 nm from a Laser Verdi G5 (Coherent, Inc.) was used to obtain the Raman spectra. Scanning electron microscopy (SEM) was conducted using an SEM-FEG scanning electron microscope (HR Inspect F50, FEI) at the Laboratory of Electron Microscopy, National Laboratory of Nanotechnology, Campinas, Brazil. For high-resolution transmission electron microscopy (HRTEM), a JEOL JEM-2100 transmission electron microscope operating at 200 kV was used. X-ray photoelectron spectroscopy (XPS) was performed using a K-Alpha XPS instrument from Thermo Fisher Scientific, featuring Al Kα emission, a vacuum of <10^–8^ mbar, and charge compensation. The spectra were collected with energy resolutions of 200 and 50 eV, using an incident radiation spot size of 400 μm in diameter. Twenty scans were performed to ensure high statistical accuracy. The optical properties of the NaNbO_3_ and Pt-NaNbO_3_ samples were analyzed by ultraviolet–visible (UV–vis) diffuse reflectance spectroscopy (DRS) with a UV–vis-NIR spectrophotometer Cary 5000 Series (Agilent Technologies), recorded from 200 to 800 nm region. Mott–Schottky plots were obtained with an AC amplitude of 10 mV and frequencies of 10, 100, and 1000 Hz in the dark. The electrochemical apparatus was the same as that used for the photoelectrochemical experiments described below.

### 2.4 Photoelectrochemical (PEC) Investigation

NaNbO_3_ and Pt-NaNbO_3_ were deposited onto the FTO substrates. Initially, suspensions of each material at a concentration of 2.5 mg mL^–1^ in a mixture of acetone and ethylene glycol (1:1) were prepared and sonicated for 90 min. The resulting suspensions were drop-cast onto the FTO substrate and air-dried, with a deposition area delimited by a Kapton tape mask measuring 1.0 × 1.0 cm^2^ on a hot plate at 100 °C. The depositions were repeated 3 times with 100 μL of the suspension. Then, the films were calcined at 300 °C for 1 h with a heating rate of 10 °C min^–1^. Electrochemical measurements were conducted using a three-electrode setup connected to a potentiostat/galvanostat (μAutolab III). The catalyst films served as working electrodes, Ag/AgCl was used as the reference electrode, and a Pt wire acted as the counter electrode. Photoelectrochemical experiments were conducted in an aqueous solution of 25% triethanolamine, degassed with argon for 20 min. Chopped-light chronoamperometry was conducted at potentials of 0, 0.2, and 0.4 V. Linear sweep voltammetry was performed with a scan rate of 0.05 V s^–1^. A solar simulator emitting a filtered A.M. 1.5G spectrum with a power intensity of 100 mW cm^–2^ was employed as the light source. The intensity was measured using a Newport model 842-PE power meter coupled to a light intensity detector (818-P-001–12).

### 2.5 Hydrogen Generation

The hydrogen generation experiments were conducted in a closed quartz cell (37 mL). For each experiment, 6 mg of NaNbO_3_ or Pt-NaNbO_3_ samples were placed in 20 mL of an aqueous solution containing 25% TEOA (as a hole scavenger). The cell was degassed with Ar for 20 min to remove dissolved oxygen. A Peschl Ultraviolet photoreactor with a UV medium-pressure lamp of 150 W (Heraeus-Noblelight) was used as the light source[Fn fn1]. A gas chromatograph (Micro GC Fusion, argon as the carrier gas, series number 70159886) was used to analyze the generation of H_2_. During the irradiation process, the reaction system was stirred with a magnetic stirrer. Each reaction lasted 4 h; all measured H_2_ amounts were the average of three samples.

## 3 Results and Discussion

### 3.1 Characterization

The diffractogram of the NaNbO_3_ sample before calcination (Figure 1a) is consistent with the structure of Na_2_Nb_2_O_6_·*x*H_2_O (ICSD 55415), commonly called Sandia Octahedral Molecular Sieves (SOMS). Upon calcination at 700 °C, only peaks corresponding to the orthorhombic NaNbO_3_ (ICSD 142291) crystal structure are observed. Although orthorhombic NaNbO_3_ resembles the SOMS, a notable distance reduction results in the disappearance of the cavities. As a result, the Na_2_Nb_2_O_6_·*x*H_2_O structure contains a significantly higher number of cavities, whereas orthorhombic NaNbO_3_ presents a more compact arrangement. This result is evidenced by the significant reduction in the surface area, which decreases from 56.00 m^2^ g^–1^ for the SOMS to 12.10 m^2^ g^–1^ for the orthorhombic NaNbO_3_ perovskite, accompanied by a decrease in the total pore volume (Table S1). The phase transition that occurs during the calcination process was assessed by TG and DSC analyses. Approximately 19% of the overall mass loss up to 260 °C is observed from the TG analysis (Figure S1). The DSC curve (Figure S2) shows the first event in the same range, from 180 to 300 °C, which is endothermic and consistent with the water loss process. A second event is observed from 430 to 500 °C (Figure S2), indicating an exothermic peak without any substantial mass loss (Figure S1), which can be attributed to a phase transition. Similar behavior was observed in our previous work,^22^ where we concluded that the material initially dehydrates, releasing water molecules. Then, the open structure transitions into a more compact orthorhombic phase of NaNbO_3_. However, it is essential to highlight the importance of the synthesis conditions on the obtained material. In our previous paper, the produced SOMS material resulting from microwave-assisted hydrothermal synthesis exhibited a surface area of 10.00 m^2^ g^–1^,^22^ which is significantly lower than the value reported here for the SOMS. We attribute this difference to the use of NbCl_5_, which undergoes a slow hydrolysis process to form Nb–O–Nb sites.

**Figure 1:**
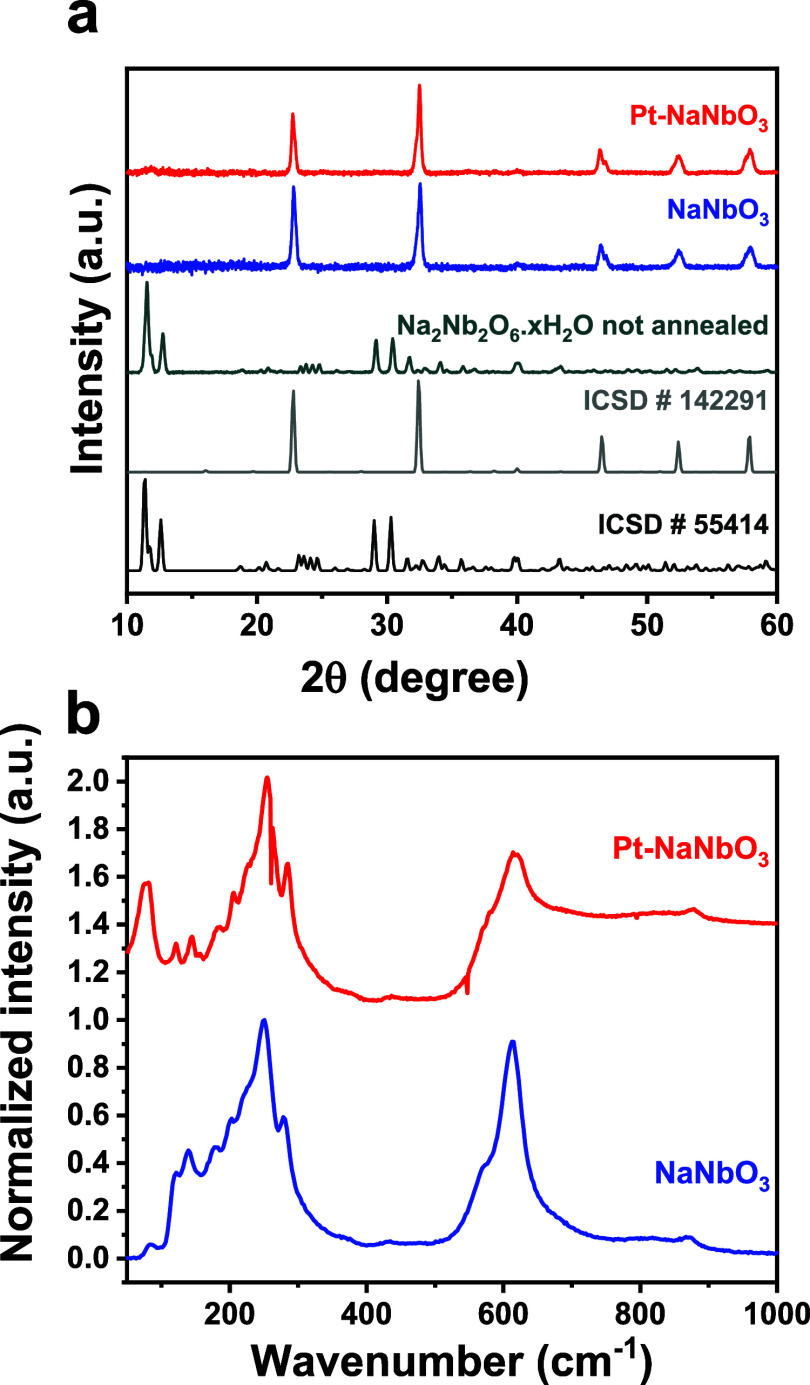
XRD patterns (a) and Raman spectra (b) of NaNbO_3_ and Pt-NaNbO_3_.

The incorporation of Pt did not significantly alter the position of the XRD peaks in the diffractogram of the resulting material (Figure 1a). Notably, the ratio of the (110) and (100) peaks at 32.4° and 22.7° remains consistent between the ICSD pattern and the NaNbO_3_ sample; on the other hand, the Pt-NaNbO_3_ sample displays an increase in the intensity of the (110) peak relative to the (100) peak (Table S2), suggesting that platinum incorporation may have induced modifications in the NaNbO_3_ crystal structure. This observation indicates that the (110) crystal plane might serve as the preferential growth plane in this sample. Also, the average crystallite sizes of the synthesized samples, estimated using the Scherrer equation^23^ with a shape factor of 0.9, yielded values of 26.8 nm for annealed NaNbO_3_ and 29.7 nm for Pt-NaNbO_3_. The modification of NaNbO_3_ with Pt also affects the surface area (Table S1). The surface area of Pt-NaNbO_3_ (17.00 m^2^ g^–1^) increased compared to NaNbO_3_. This result could further evidence the incorporation of Pt into the semiconductor.

The Raman spectrum of NaNbO_3_ (Figure 1b) displays the characteristic bands of an orthorhombic crystal structure,^22,24,25^ corroborating the XRD findings. The NaNbO_3_ and Pt-NaNbO_3_ materials show a band at 620–630 cm^–1^ and another in the 520–580 cm^–1^ region, corresponding to the Nb–O bond distances. The Raman bands at ∼870, ∼430, and ∼375 cm^–1^ are attributed to the antisymmetric stretching mode of the Nb–O–Nb bond and the associated bending modes of the Nb–O–Nb bond, respectively. The bands at 197–300 cm^–1^ are related to the bending of the NbO_6_ octahedra, and, finally, those below 200 cm^–1^ are associated with the translational mode of Na^+^.^26,27^ However, the Pt-NaNbO_3_ sample shows a slight increase in peak intensity between 200 and 350 cm^–1^, along with an elevated baseline above 600 cm^–1^. These results suggest that the Pt induces a SERS (surface-enhanced Raman spectroscopy) effect in the Raman spectra.

The morphology of Na_2_Nb_2_O_6_·*x*H_2_O, analyzed through SEM images, reveals an aggregate of wire-like structures (Figure 2a), indicating that crystal growth occurs during the microwave-assisted hydrothermal treatment via a dissolution-recrystallization mechanism, which gives rise to single crystals as can be seen in the lattice fringes observed in the HRTEM (Figure S3a,b).^22,28^ However, after annealing, there is a noticeable increase in wire thickness, accompanied by the appearance of irregular edges (Figure 2b), which is further confirmed by the TEM image of NaNbO_3_ (Figure S3c). In our previous study,^22^ we similarly synthesized NaNbO_3_ using a microwave-assisted method, but at higher temperatures (180 °C) and shorter reaction times (15 min), resulting in faster reaction kinetics than those observed in the current study. As a result, the diameter of the wires was 250–500 nm. In the current work, the slower reaction kinetics produce wire-like structures with smaller diameters, as both the NaNbO_3_ samples before and after annealing exhibit diameters of only a few nanometers. This difference in particle growth kinetics led to materials with larger surface areas, which undoubtedly influenced their efficiency. The crystallinity of NaNbO_3_ is confirmed by the distinct lattice fringes observed in the HRTEM image (Figure 2c). From the FFT of the HRTEM image, three interplanar distances could be identified: 3.90 Å (100), 2.76 Å (110), and 1.95 Å (200), corroborating the XRD results for NaNbO_3_.

**Figure 2:**
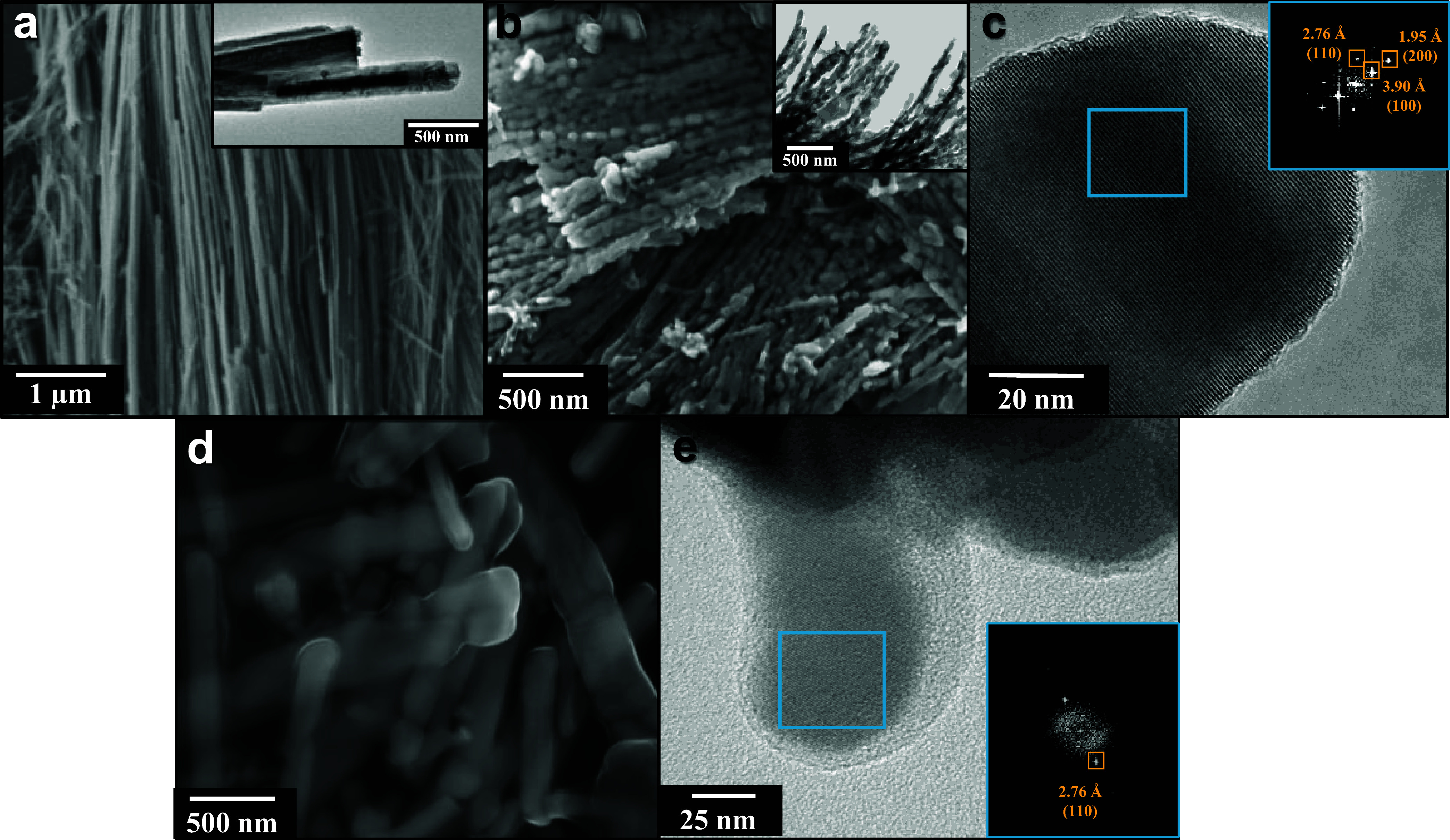
SEM image of Na_2_Nb_2_O_6_·*x*H_2_O not annealed (a); SEM (b) and TEM (c) images of NaNbO_3_; and SEM (d) and TEM (e) images of Pt-NaNbO_3_.

The addition of Pt into NaNbO_3_ (Figure 2d) led to an enlargement of the wires with the formation of a smooth surface, which can be ascribed to the formation of a Pt species at the surface of the NaNbO_3_. However, there was no evidence of Pt nanoparticle deposition on the one-dimensional NaNbO_3_ structures from the SEM images. Similarly, no Pt particles were detected in the TEM images (Figure 2e). The material maintained its high crystallinity, as indicated by the clear lattice fringes in the HRTEM images (Figure 2e). Interestingly, the FTT analysis of the HRTEM region revealed only one lattice spacing, corresponding to the (110) crystal plane of NaNbO_3_, which aligns with the preferential growth plane identified in the XRD pattern (Figure 1a). Therefore, considering the changes in the crystallinity observed in the XRD and HRTEM, the SERS effect observed in the Raman spectra, and the incapacity of detecting the Pt through TEM, we hypothesize that the Pt has been incorporated as a species, whose nature we could not identify, and cannot be detected using the transmission electron microscope, which we had access to.

The high-resolution O 1s spectra of the synthesized materials (Figure 3a) show two main peaks, which are further decomposed into three components. The first peak, with a maximum intensity ranging from 529.98 to 530.58 eV, according to the photocatalyst, corresponds to the coordinated oxygen species (O^2^_latt_^–^). The second peak, located at binding energy ranging from 531.28 to 532.68 eV, is associated with the formation of O^–^ and O_2_^2–^ species adsorbed on the material’s surface due to the calcination step (O_ads_). The presence of O_ads_ species may be related to oxygen vacancies.^8,17^ At around 534 eV, the third peak is assigned to surface oxygen connected by double bonds (O_db_). The main peak of the O 1s shifts ∼0.6 eV to a higher binding energy after Pt incorporation. This usually suggests that the electron density around O decreases, potentially due to electron withdrawal by Pt or formation of a Pt–O interaction.^29^ The high-resolution XPS spectra of Nb 3d (Figure 3b) display two peaks at approximately 207 and 210 eV, corresponding to the Nb 3d_5/2_ and Nb 3d_3/2_ doublets, respectively, consistent with the Nb­(V) in the [NbO_6_] octahedra.^30,31^ After Pt incorporation, a slight shift (∼0.8 eV) toward higher binding energies is observed, which can be attributed to electronic interactions between the Nb and Pt species. This shift indicates a subtle redistribution of electron density, likely due to the incorporation of oxidized Pt species into or onto the NaNbO_3_ surface.^32^ Finally, the two Pt 4f peaks of Pt-NaNbO_3_ (Figure 3c) at 73.58 and 76.88 eV, were assigned to Pt^4+^ 4f_7/2_ and 4f_5/2_, respectively.^33^ The peaks exhibit low intensity, which is attributed to the low content of the element, estimated at 0.24 atom % (Figure S4). This amount is lower than the proportion added during the preparation of the material, indicating that the method failed to ensure complete deposition of Pt. The presence of Pt predominantly in the Pt^4+^ oxidation state is consistent with PtO_2_-like species highly dispersed on the NaNbO_3_ surface. Although the synthesis conditions are not expected to yield PtO_2_ directly,^34,35^ XPS revealed only Pt^4+^ species, which can be attributed to the strong surface sensitivity of the technique and the oxidation of highly dispersed Pt at the outer layers. In contrast, metallic Pt^0^ formed during photodeposition may remain undetectable beneath the oxidized surface. The absence of detectable Pt nanoparticles in TEM (Figure 2) and XRD (Figure 1a), combined with the XPS results, further supports the hypothesis of the formation of Pt species. It is worth noting that, under photocatalytic conditions, photogenerated electrons can progressively reduce Pt^4+^ to Pt^0^, which is the active form responsible for promoting H_2_ evolution.^36^

**Figure 3:**
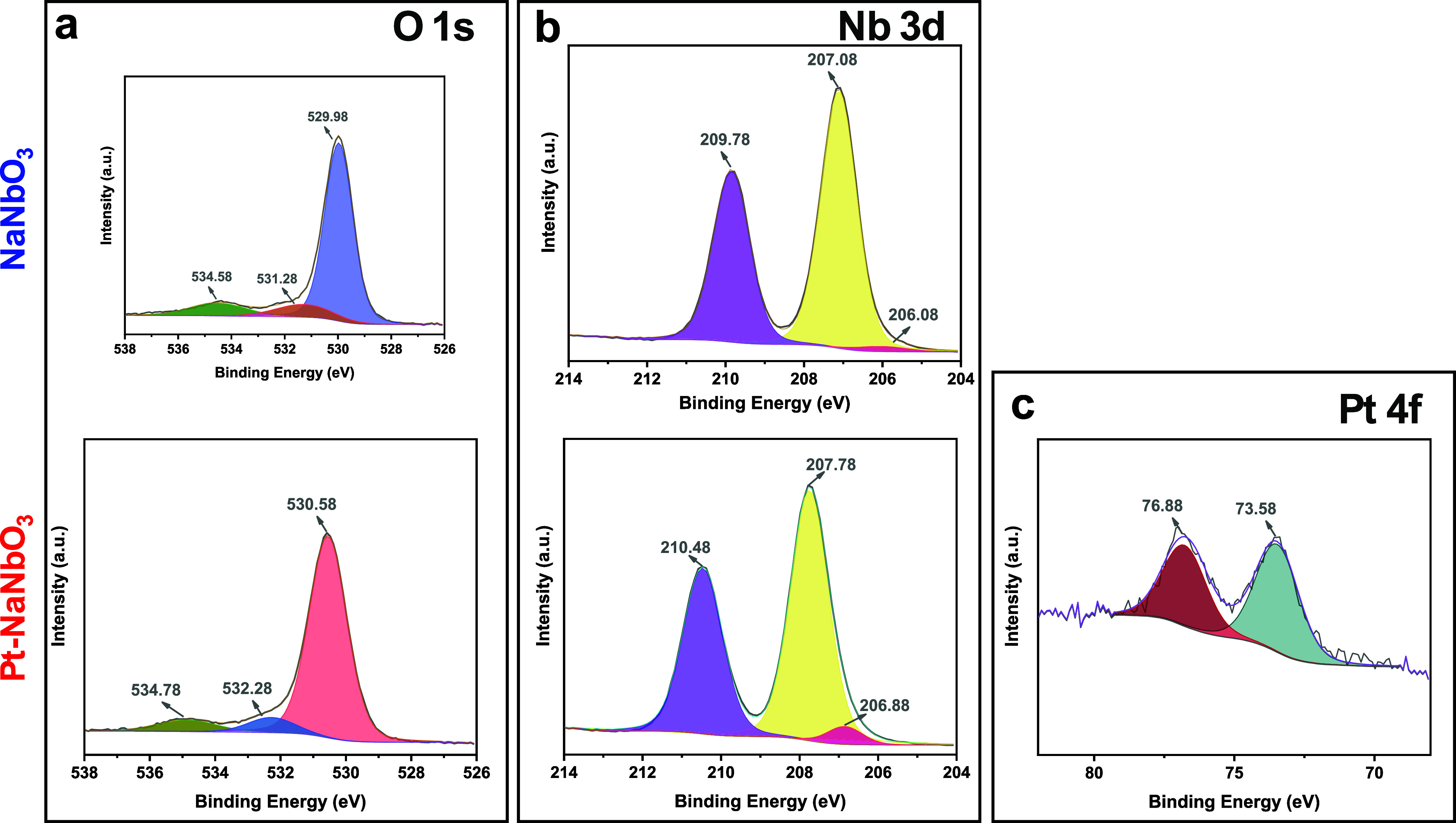
High-resolution XPS spectra of 1 s of (a), 3d of (b), and 4f of (c) of NaNbO_3_ and Pt-NaNbO_3_.

Additionally, in contrast to the microscopy analyses (Figure 2), which revealed no Pt nanoparticles in the examined regions, the XPS analysis confirms the presence of Pt in the materials. This reinforces the hypothesis that Pt particles may be distributed at the molecular level as a compound.

The optical properties investigated using UV–vis diffuse reflectance spectroscopy (DRS) reveal that the materials exhibit outstanding absorption in the UV (NaNbO_3_) and some absorption in the visible range (Pt-NaNbO_3_), as shown in Figure S5a. The bandgap energies of the NaNbO_3_ and Pt-NaNbO_3_ samples were estimated using the Kubelka–Munk function from DRS data (see the SI file; Figure S5b).

The bandgap energies can be estimated by considering the direct injection (Table 1). The bandgap energy results obtained for pristine NaNbO_3_ align with values previously published in the literature.^20,37^ The Pt-NaNbO_3_ junction exhibited a significant increase in the bandgap energy, an unexpected result. According to previous studies, incorporating platinum typically leads to a decrease in bandgap energy.^17,38^ We attribute this observation to the eventual intercalation of Pt into the NaNbO_3_ structure, as suggested by the XRD and HRTEM data.

**Table 1:** Calculated Direct and Indirect Bandgap Energies, Flat Band Potential, and Estimated Valence Band Potential of the Catalysts

material	*E*_g direct_ (eV)	*V*_fb_ (V)	VB (V)
NaNbO_3_	3.31	–0.93	2.38
Pt/NaNbO_3_	3.75	–0.68	3.07

To determine the flat band potentials, Mott–Schottky plots were taken using the relation as follows (eq 1)^39^

1 $$\frac{1}{C^{2}}=\left(\frac{2}{e\varepsilon \varepsilon_{0}N_{d}}\right)[V_{a}-V_{\mathrm{fb}}-\frac{\mathrm{kT}}{e}]$$

where *C* is the space charge layer capacitance, *e* is the electron charge, ε is the dielectric constant, ε_0_ is the permittivity of vacuum, *N*_d_ is the charge carrier density, *V*_a_ is the applied potential, and *V*_fb_ is the flat band potential. The flat band potential *V*_fb_ was determined by taking the *x*-intercept of a linear fit to the Mott–Schottky plot, $\frac{1}{C^{2}}$ , as a function of applied potential (*V*_a_) when the frequency is 1000 Hz, Figure 4a; Table 1 summarizes the *V*_fb_ calculated. To confirm the obtained result, *V*_fb_ was also estimated from the Mott–Schottky plot obtained using frequencies of 10 and 100 Hz (Figure S6 and Table S3). The results show that all materials exhibit positive slopes, indicating they are n-type semiconductors. For pristine NaNbO_3_, the *V*_fb_ is −0.93 V vs Ag/AgCl (3 M KCl). The modifications with Pt induced a shift of flat potential toward a less negative value (−0.68 V, see Table S3). These results, combined with the DRS, Raman, and HRTEM data, reinforce the hypothesis of an intercalation of Pt into the NaNbO_3_ structure.

**Figure 4:**
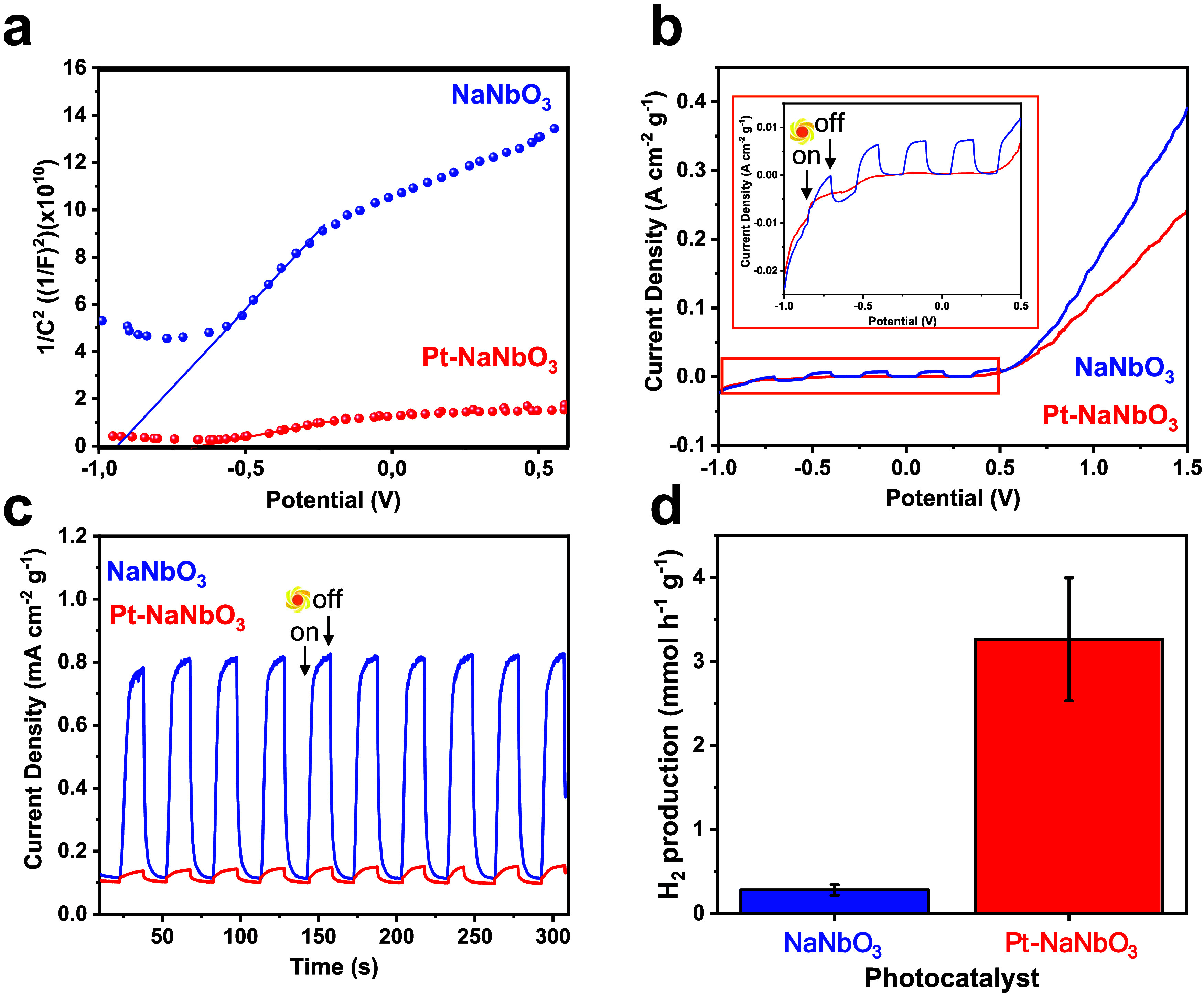
Mott–Schottky plots of NaNbO_3_ and Pt-NaNbO_3_ collected at a frequency of 1000 Hz using a Ag/AgCl reference electrode in 3 M KCl (a). Chopped-light liner sweeps voltammetry using NaNbO_3_ and Pt-NaNbO_3_ as working electrodes at a scan rate of 10 mV s^–1^ with an inset highlighting the photocurrents measured between −1.0 and 0.5 V, under solar simulator A.M. 1.5G irradiation (b). Chopped-light chronoamperometry using NaNbO_3_ and Pt-NaNbO_3_ as working electrodes under an applied bias of 0.4 V and solar simulator A.M. 1.5G irradiation (c). Hydrogen evolution rate under a 150 W Hg medium-pressure lamp (d).

Additionally, the density of the charge carriers is substantially dependent on the modification of NaNbO_3_. The *N*_*d*_ can be derived from eq 1 by the following equation

2 $$N_{d}=\frac{2}{\varepsilon \varepsilon_{0}e}\frac{dE}{d\frac{1}{C^{2}}}=\frac{2}{\varepsilon \varepsilon_{0}e}\frac{1}{\mathrm{slope}}$$

The plots in Figure 4a can be used to extract the charge carrier density from the slopes. Table S3 summarizes the values found. It can be observed that pristine NaNbO_3_ has a gentler slope compared to Pt-NaNbO_3_, indicating a higher density of charge carriers. This property influences the photocatalytic efficiency, as discussed in the next section. Additionally, band alignment plays a crucial role in determining the photoactivity. By combining the results from DRS and Mott–Schottky plots, we can propose the band alignment of the photocatalysts. First, it is considered that the *V*_fb_ is approximately equal to the VB potential; second, it is assumed that only one electron makes part of the charge injection from the VB to the CB. Thus, the VB potential can be estimated, and the resulting values are presented in Table 1. Therefore, it was estimated that both materials exhibit suitable potential to perform both HER and OER. However, the bandgap energies required to produce the photogenerated charges differ significantly (Table 1).

### 3.2 Photoelectrochemical Experiments

The photoelectroactivity of each electrode was determined by measuring the current density during the OER as AM1.5G simulated solar light was irradiated on the front side of the film. Figure 4b exhibits the chopped-light linear sweep voltammetry obtained. It can be observed that the photoresponse of the photoanodes is not rapid under chopped-light conditions. NaNbO_3_ exhibited an increase in current upon irradiation and a low onset potential. The observed photocurrents can also be attributed to differences in the density of charge carriers (Table S3).

Chopped-light chronoamperometry (Figure 4c) shows the better photoresponse capacity of the photoanodes. The photocurrents produced by pristine NaNbO_3_ are approximately five times higher than those of Pt-NabO_3_. Chopped-light chronoamperometry at other potentials was also obtained, showing the same trend (Figure S7). Therefore, we can conclude that modifying NaNbO_3_ with Pt failed to increase the activity of the photoanodes in the direction of the OER reaction. This can be attributed to the significant increase in bandgap energy and a corresponding decrease in charge carrier density.

### 3.3 Hydrogen Production

The photocatalytic activity of the materials was investigated through a hydrogen evolution reaction under UV-vis light (Figure 4d). NaNbO_3_ produced ∼0.28 mmol h^–1^ g^–1^ of H_2_. According to the literature, the hydrogen evolution rates for pure NaNbO_3_ are typically around 0.25 and 0.40 mmol h^–1^ g^–1^.^17,40^

The Pt-NaNbO_3_ sample produced 1.26 mmol h^–1^ g^–1^ of hydrogen, which might be ascribed to the role of Pt as an electron sink under no applied bias conditions. In this condition, photogenerated electrons flow from NaNbO_3_ to the Pt atoms at the surface, where H^+^ is converted into H_2_. From the XPS analysis, only Pt^4+^ species were detected in the as-prepared material; however, these can be photoreduced to metallic Pt during the photocatalytic reaction, thereby generating active sites that further enhance H_2_ production.^36^ Although no directly comparable material has been reported in the literature, Yang and co-workers produced nitrogen-doped NaNbO_3_ coupled to Pt by conventional hydrothermal treatment at 180 °C for 2 h, achieving a hydrogen production rate of 1.4 mmol h^–1^ g^–1^.^17^ The marked enhancement observed for Pt-NaNbO_3_, despite the apparent absence of metallic Pt in the sample, can thus be rationalized by this in situ reduction pathway, which has also been described for other oxide-supported Pt systems.

## 4 Conclusions

This study demonstrated that modifying NaNbO_3_-based photocatalysts with Pt induced a significant enhancement in photocatalytic hydrogen production. The synthesis of NaNbO_3_ involved a transition from Na_2_Nb_2_O_6_·*x*H_2_O to an orthorhombic NaNbO_3_ structure, confirming the successful preparation of the material. The introduction of Pt into the NaNbO_3_ structure did not cause significant changes in the material’s crystalline structure, but it played a key role in enhancing photocatalytic activity. The Pt-NaNbO_3_ composite demonstrated the highest hydrogen production among the tested materials, supporting the idea that Pt facilitates charge separation and enhances photocatalytic performance. This enhancement can be attributed to the presence of Pt as a cocatalyst, which aids electron trapping, improves electron transfer efficiency, reduces recombination losses, and promotes the reduction of protons to hydrogen. Therefore, we were able to produce a photocatalyst for hydrogen generation with high efficiency using a strategy that is less energy and time-consuming than the previous reported materials.

## Supplementary Material



## Footnotes

1 Spectrum available at https://www.noblelight.com/en/etc/products_and_solutions/uv_lamps_and_systems/uv_lamps/uv_medium_pressure_lamps/uv_medium_pressure_lamps.html#tabs-43875-3

## Notes

Thermal analysis, XPS survey spectra, DRS data treatment, and additional photoelectrochemical data (PDF)
