# Supplementary material for: Microwave-Assisted Synthesis of Pt-Modified NaNbO3 Nanowires for Enhanced Photocatalytic Hydrogen Production
Source: ACS Omega. 2025 Dec 5;10(49):60641–8. doi: 10.1021/acsomega.5c08475 (PMC12713482; doi:10.1021/acsomega.5c08475)
Supplement: Supplementary file 1 [file ao5c08475_si_001.pdf]

Supporting Information

Microwave-Assisted Synthesis of Pt-Modified $NaNbO_3$ Nanowires for Enhanced Photocatalytic Hydrogen Production

Marcos R. S. Vicente,[a] Priscila H. Palharim,[a] Gabriela T. M. Xavier,[a] Wagner A. Carvalho,[a] Hynd Remita[b] and Juliana S. Souza

[a]*Centro de Ciências Naturais e Humanas, Universidade Federal do ABC, 09210-580, Santo André, SP, Brazil*

[b]*Université Paris-Saclay, UMR 8000 CNRS, Institut de Chimie Physique, Orsay, 91405 France*

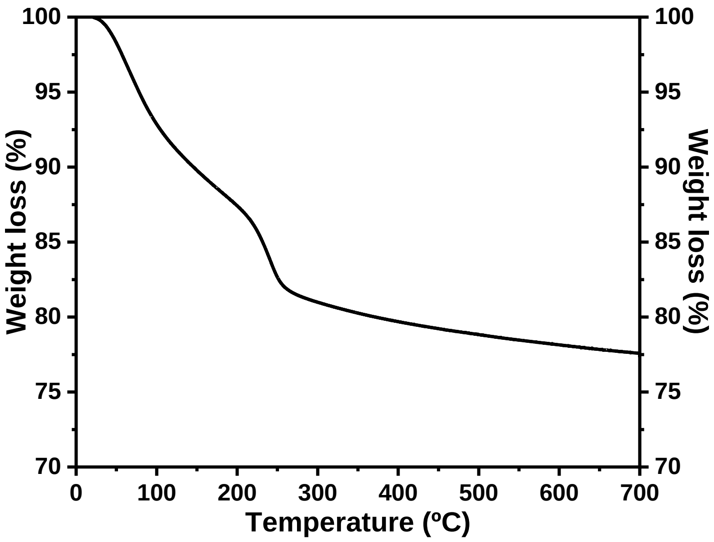

Figure S1. Thermogravimetric analysis of simulated annealing conditions of $NaNbO_3$.

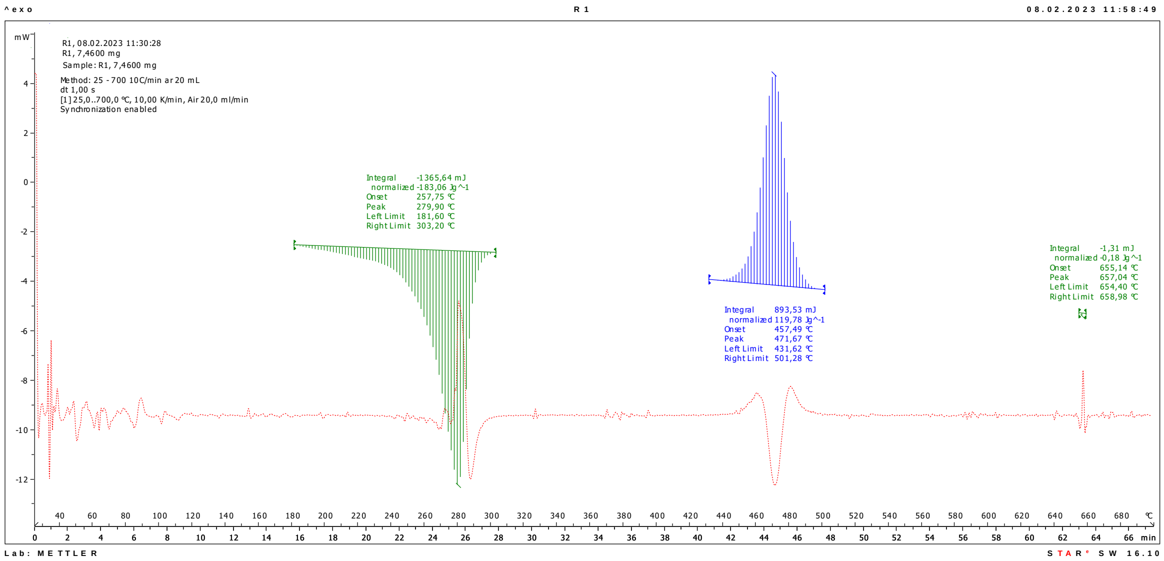

Figure S2. Differential scanning calorimetry (DSC) analysis of simulated annealing conditions of $NaNbO_3$.

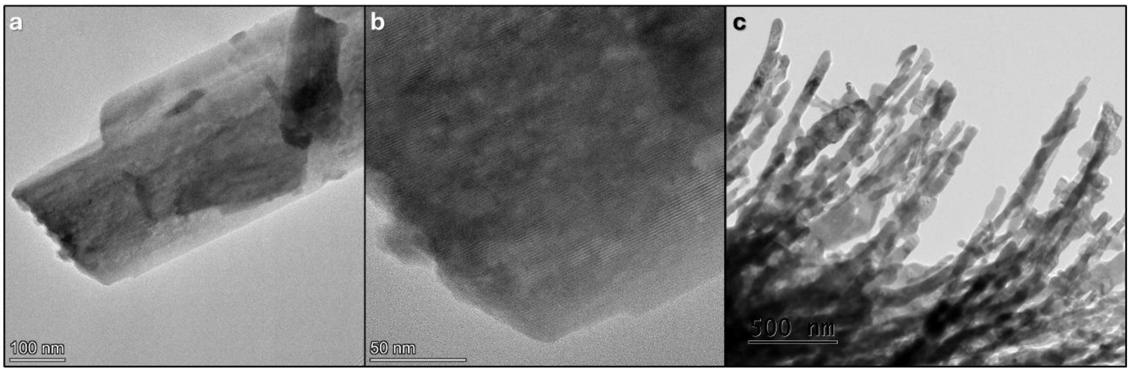

Figure S3. a) TEM and b) HRTEM images of $Na_2Nb_2O_6.xH_2O$ not annealed; c) TEM image of $NaNbO_3$.

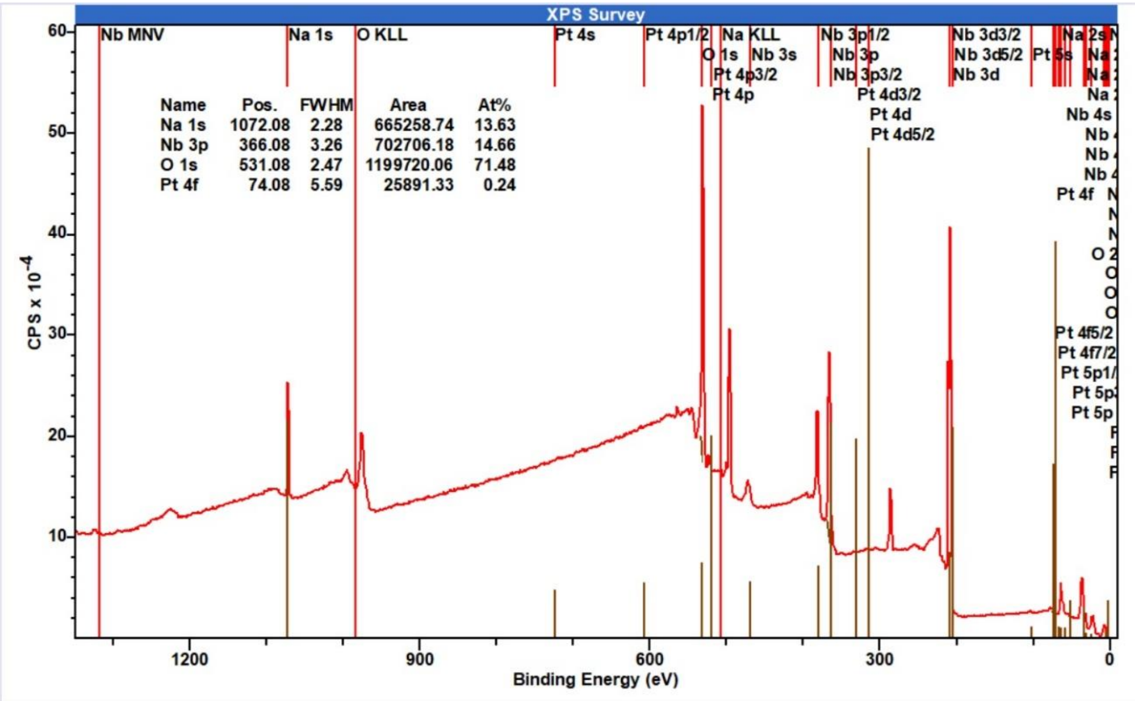

Figure S4. Atomic percentage of the elements found in the Pt-$NaNbO_3$ sample.

Band gap energy calculation

Kubelka Munk Function (Equation S1)

$$F(R) = K/S \qquad \text{Equation S1}$$

where K is the molar absorption coefficient (Equation 2) and S is the scattering factor (Equation 3):

$$K = (1 - R)^2 \qquad \text{Equation S2}$$

$$S = 2R \qquad \text{Equation S3}$$

R is the reflectance of the material (Equation 4):

$$R = \%R/100 \qquad \text{Equation S4}$$

The combination of Equations 2, 3 and 4 gives Equation 5:

$$F(R) = (1 - R)^2/2R \qquad \text{Equation S5}$$

By plotting the $[F(R) \times hn]^n$ vs hn it is possible to estimate the direct band gap value, where n is equal to 2 for direct injection and equal to ½ for indirect injection (Figure S6).

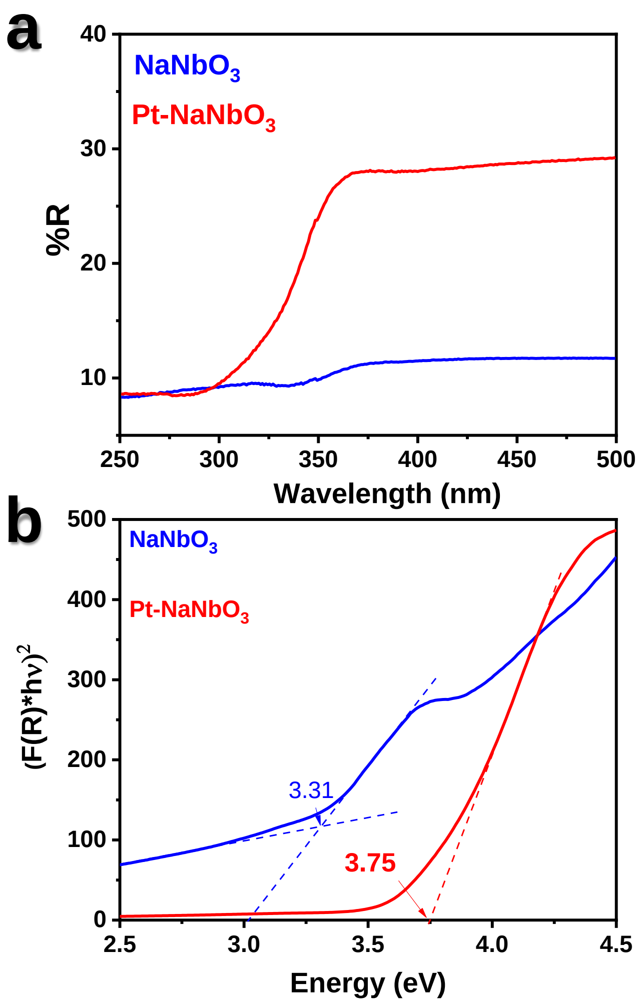

Figure S5. %Reflectance spectra (a), direct band gap plot (b) of $NaNbO_3$ and Pt-$NaNbO_3$.

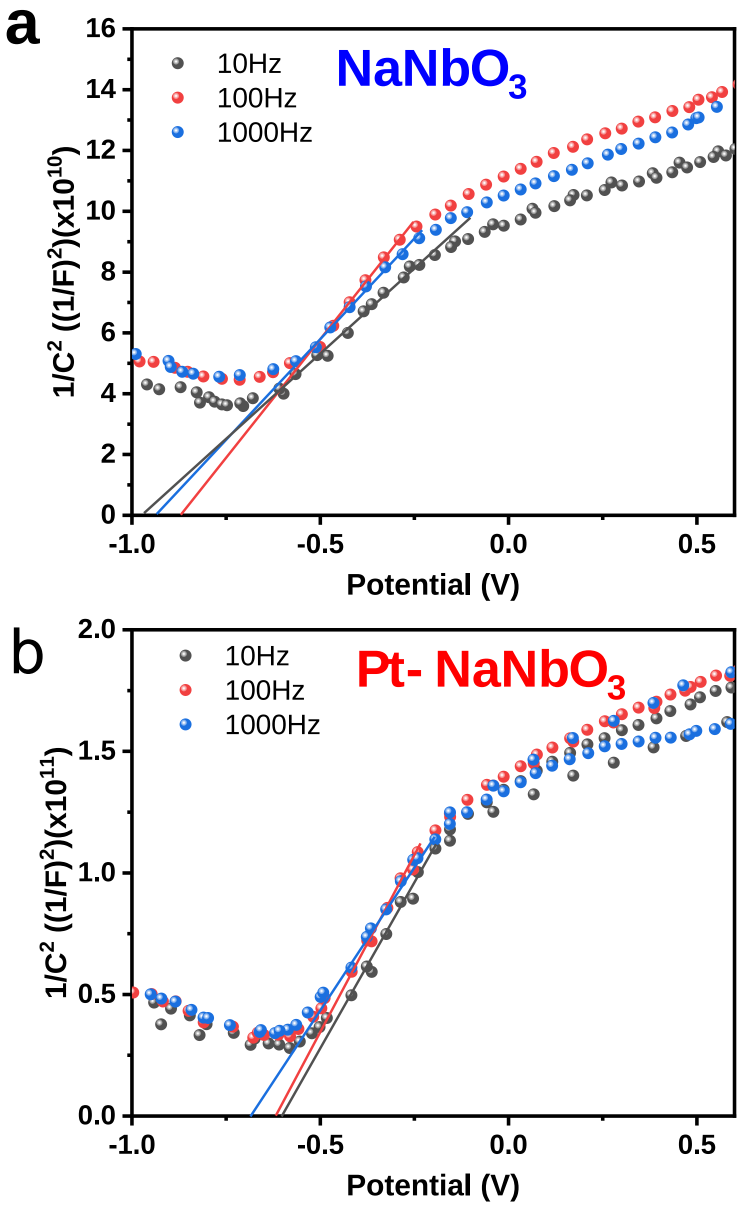

Figure S6. Mott-Schottky plots obtained using the $NaNbO_3$ (a) and Pt-$NaNbO_3$ (b) as working electrodes at frequencies of 10, 100 and 1000 Hz.

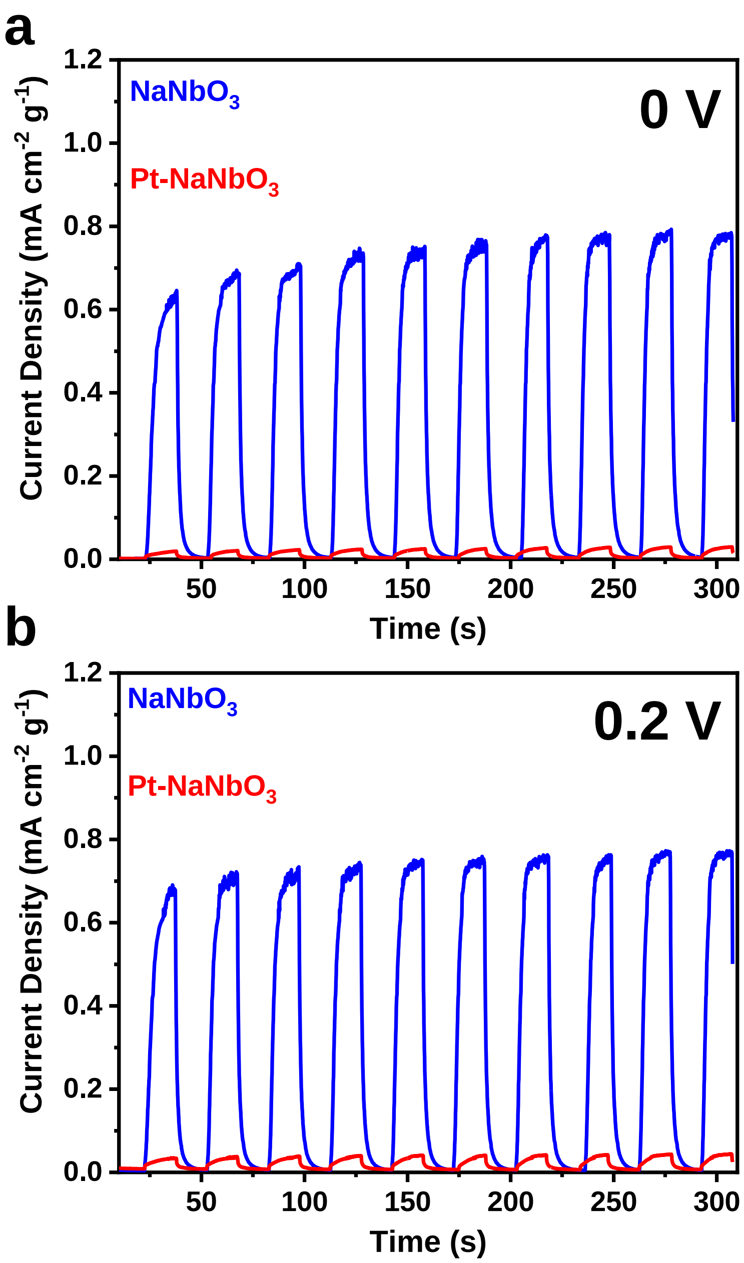

Figure S7. Chopped-light chronoamperometry experiments using $NaNbO_3$ and Pt-$NaNbO_3$ as working electrodes, Pt wire as counter electrode, Ag/AgCl 3M KCl as reference electrode, and an aqueous solution of TEOA 25% as electrolyte, under applied bias of 0V (a) and 0.2 V (b).

Table S1. Surface area and volume of pores of the synthesized materials.

**Material**	**Surface area S_{BET} (m^2/g)**	**Total pore volume (cm^3/g)**
$Na_2Nb_2O_6.xH_2O$ (SOMS)	56	0.21
$NaNbO_3$	12	0.078
Pt-$NaNbO_3$	17	0.10

Table S2. (110)/(100) peak ratio of the synthesized materials.

Material	(110)/(100) peak ratio
ICSD 142291	1.12
$NaNbO_3$	1.03
Pt-$NaNbO_3$	1.46

Table S3. Calculated slope, intercept and V_{fb} values from the linear fit of the Mott-Schottky plots obtained at 10, 100 and 1000 Hz. Reference electrode Ag/AgCl 3M KCl.

Material	10 Hz			100 Hz			1000 Hz		
	Slope	Intercept	V_{fb}(V)	Slope	Intercept	V_{fb}(V)	Slope	Intercept	V_{fb}(V)
$NaNbO_3$	1.125E11	1.095E11	-0.97	1.608E11	1.377E11	-0.85	1.340E11	1.248E11	-0.93
Pt-$NaNbO_3$	2.761E11	1.653E11	-0.60	2.817E11	1.771E11	-0.63	2.391E11	1.627E11	-0.68
